# The Shenlian Fumai Granule attenuates Ach-CaCl_2_-induced atrial fibrillation by regulating atrial electrical and structural remodeling

**DOI:** 10.3389/fcvm.2025.1573728

**Published:** 2025-12-15

**Authors:** Youjin Kong, Lingling Xie, Zheng Ma, Zhengtian Lv, Tingting Chen, Chenxia Wu, Jin Dai, Xiaoming Xu, Miaojun Lian, Xinbin Zhou, Wei Mao

**Affiliations:** 1 The First School of Clinical Medicine, Zhejiang Chinese Medical University, Hangzhou, China; 2 Department of Cardiology, The First Affiliated Hospital of Zhejiang Chinese Medical University (Zhejiang Provincial Hospital of Chinese Medicine), Hangzhou, China; 3 Zhejiang Key Laboratory of Integrative Chinese and Western Medicine for Diagnosis and Treatment of Circulatory Diseases, Zhejiang Hospital (Affiliated Zhejiang Hospital, Zhejiang University School of Medicine), Hangzhou, China; 4 Department of Cardiology, Zhejiang Hospital (Affiliated Zhejiang Hospital, Zhejiang University School of Medicine), Hangzhou, China

**Keywords:** traditional Chinese medicine, atrial fibrillation, Shenlian Fumai Granule, network pharmacology, atrial remodeling

## Abstract

**Background:**

Preliminary clinical studies indicate that Shenlian Fumai Granule (SLFM) reduces the incidence of arrhythmias following radiofrequency ablation in patients with atrial fibrillation (AF) and improves clinical symptoms. This study investigates the preventive effects and underlying mechanisms of SLFM on AF, providing robust evidence to support its clinical application.

**Methods:**

The potential therapeutic effects of SLFM on AF were investigated using network pharmacology. Subsequently, High Performance Liquid Chromatography-Mass Spectrometry (HPLC-MS) was employed for further analysis of SLFM, while molecular docking was utilized to validate potential targets. The impact of SLFM on the electrophysiology of rat hearts was examined during Langendorff perfusion through electrical mapping. AF in rats was induced using Ach (66 μg/mL) and CaCl_2_ (10 mg/kg), with a dose of 0.1 mL/100 g injected into the tail vein for a duration of 5 weeks. SLFM was administered daily at doses of 3.645 and 7.29 g/kg, with amiodarone (0.18 g/kg) serving as the positive control. The effects of SLFM on AF were assessed through electrocardiography, echocardiography, ELISA, and histopathological analysis.

**Results:**

A total of 107 active compounds were extracted, and their relationships with Traditional Chinese Medicines (TCMs) and protein targets were analyzed. The intersection of potential TCM target genes and genes related to AF identified four significant genes. Gene Ontology (GO) and Kyoto Encyclopedia of Genes and Genomes (KEGG) analyses indicated that these targets may be involved in various pathways associated with AF. Furthermore, SLFM was found to prolong the atrial effective refractory period in a concentration-dependent manner, suggesting a potential anti-atrial arrhythmia effect. In a rat model of AF induced by Ach-CaCl_2_, SLFM treatment significantly reduced markers related to atrial electrical remodeling, including the induction rate and duration of AF, while also alleviating fibrosis and the upregulation of Connexin 43 (Cx43) expression.

**Conclusion:**

This study demonstrated that SLFM plays a critical role in suppressing atrial electrical and structural remodeling induced by Ach-CaCl_2_, thereby further reducing susceptibility to AF. Consequently, SLFM may hold potential as a therapeutic agent for the treatment of AF.

## 1 Introduction

Atrial fibrillation (AF) is a common clinical condition characterized by rapid atrial arrhythmia and represents the most common sustained cardiac arrhythmia worldwide. Its incidence and prevalence increase significantly with age, posing a growing global public health challenge (1). According to the 2024 ESC Guidelines, an estimated 59 million people were living with AF in 2017, reflecting a twofold increase since 1990, with the age-standardized prevalence now exceeding 9% in individuals aged 80 years or older (2). This upward trend is similarly observed in China (3), underscoring the escalating burden of the disease. AF notably impairs patients' quality of life and elevates the risk of mortality and morbidity associated with stroke, heart failure, chronic kidney disease, and dementia (4). The cornerstone of AF management involves anticoagulation therapy alongside heart rate and rhythm control. When pharmacological interventions are insufficient, ablation and pacemaker implantation serve as primary alternatives (2). However, the limited effectiveness and adverse reactions of antiarrhythmic drugs (AADs), coupled with the high cost and suboptimal long-term efficacy of ablation procedures (5–8), highlight the urgent need for alternative and more effective treatment strategies.

Traditional Chinese medicine (TCM) is based on the principles of “holistic view” and “treatment based on pattern differentiation”, considered more effective, with fewer adverse reactions and lower toxicity compared with modern medicine, is widely used in clinical practice (9). A previous study demonstrated that treatment with Shensong Yangxin capsule following radiofrequency catheter ablation for persistent AF reduced the incidence of recurrent atrial tachyarrhythmias and led to clinically significant improvements in quality of life during a 12-month follow-up in a Chinese population (10). Furthermore, AF results from the interplay of multiple factors, which complicates effective treatment using single-target therapies. Consequently, the multi-target treatment approach of TCM may represent a potential option for the prevention and treatment of AF.

The Shenlian Fumai Granule (formerly known as Yiqi Fumai mixture) is a TCM formula developed by Liao Jiazhen, an expert in Traditional Chinese Medicine based in Beijing, China. This formula comprises 11 medicinal herbs, including 15 g of *Dangshen*, 9 g of *Banxia*, 10 g of *Huanglian*, 15 g of *GuiJianYu*, 15 g of *ChuanXiong*, 30 g of *DanShen*, 15 g of *ChiShao*, 15 g of *BaiShao*, 10 g of *ZhiGanCao*, 30 g of *SuanZaoRen*, and 10 g of *YuanZhi*. A clinical study demonstrated that SLFM significantly reduces the Traditional Chinese Medicine Evidence Score for ventricular premature contractions when compared to a placebo (11). *Danshen*, the monarch herb in this formula, serves multiple functions including tonifying qi, activating blood circulation, clearing heat, resolving phlegm, calming the mind, and tranquilizing the spirit. Preliminary clinical studies indicate that SLFM can decrease the incidence of arrhythmias in patients with AF following radiofrequency ablation, while also improving clinical symptoms (12). Although previous studies on Fumai-series formulas have reported electrophysiological and structural benefits in AF models (13), the specific mechanism of SLFM—with its distinct composition of 11 herbal medicines—remains unclear.

## 2 Materials and methods

### 2.1 Drug-ingredient-target network construction

The chemical constituents of each herbal component in SLFM were retrieved from the TCMSP database (https://www.tcmsp-e.com). These constituents were screened based on oral bioavailability (OB) ≥ 30% and drug-likeness (DL) ≥ 0.18. Potential targets for each constituent were predicted, and a dataset linking SLFM to its components and targets was constructed. The network was visualized using Cytoscape software (version 3.7.2).

### 2.2 Screening of key genes related to diseases

Transcriptome data related to AF were retrieved from the GEO database (accession number GSE197518), comprising left atrial appendage samples from three patients with AF and three healthy controls. Differential expression analysis was conducted using the limma package in R, with filtering based on an absolute log2 fold change > 1 and a *P*-value < 0.05.

### 2.3 Construction of PPI network related to AF genes

Utilizing the STRING database (https://cn.string-db.org/), systematically collect and integrate the interactions among genes associated with AF to construct a primary protein-protein interaction (PPI) network. Isolated protein targets that do not interact with any others should be removed from the primary network to establish a more stable secondary PPI network. Proteins in the secondary PPI network will be sorted based on the number of interactions, with targets exhibiting interactions ≥ 30 selected as core proteins, subsequently leading to the construction of a tertiary PPI network. Additionally, the MCODE plugin will be employed to identify the core modules within the tertiary PPI network, resulting in the formation of a quaternary PPI core network. All the aforementioned PPI networks will be visualized using Cytoscape software (version 3.7.2).

### 2.4 Analysis of the core targets enriched by SLFM

Utilizing the “clusterProfiler” package in R, along with the “org.Hs.eg.db” data package, we will conduct an enrichment analysis on the core target proteins associated with coronary artery disease. The “ont” parameter for the GO enrichment analysis will be set to “ALL”, and the *p*-value adjustment method will be specified as “BH”. The results of the enrichment analysis will be visualized using the “GOplot” package. Additionally, a KEGG enrichment analysis will be performed using the “enrichKEGG” function, applying a significance threshold of *p* < 0.05.

### 2.5 Molecular docking

To retrieve the three-dimensional structural data of key target proteins, access the Protein Data Bank (PDB) database (https://www.rcsb.org/). Convert the structure files of active ingredients from SDF format to PDB format using Chem 3D (19.0) software. Utilize AutoDockTools (1.5.6) to identify the active pocket positions of the target proteins and convert the PDB format to PDBQT format for subsequent molecular docking. Perform molecular docking of the target proteins with the active ingredients using AutoDock Vina, employing gradient optimization methods to identify the most suitable structural conformations, and calculate the binding affinity between the two.

### 2.6 SLFM preparation

The medicinal materials used by SLFM are all purchased from the Renowned Traditional Chinese Medicine Clinic of Zhejiang Chinese Medical University, and are prepared by the School of Traditional Chinese Medicine of the same university. SLFM is protected under patent number 201310303390.5. The initial step involves the careful selection of specific medicinal herbs, including *Dangshen* (Gansu, Lot No. 1240807X), *Banxia* (Hebei, Lot No. 240301), *ChiShao* (Inner Mongolia, Lot No. 240921), *ZhiGanCao* (Inner Mongolia, Lot No. 1240911X), *GuiJianYu* (Hunan, Lot No. 240501), and *BaiShao* (Anhui, Lot No. 240901). These herbs are first boiled with ten times the volume of water for 1.5 h, after which the mixture is filtered. This process is repeated twice more, with the second and third boils using eight times the volume of water for 1.5 h each, followed by filtration. The filtrates from all three boils are then combined, concentrated to a relative density of 1.18 at 55°C, and subsequently dried. For the herbs *DanShen* (Shandong, Lot No. 240902), *Huanglian* (Sichuan, Lot No. 1240510X), *YuanZhi* (Shanxi, Lot No. 1240814X), *ChuanXiong* (Sichuan, Lot No. 1240824X), and *SuanZaoRen* (Shandong, Lot No. 241025), the first boiling is conducted with ten times the amount of 60% ethanol for 2.0 h, followed by filtration. The second boiling employs nine times the amount of 60% ethanol for another 2.0 h, after which the filtrates from both boils are combined and filtered again. The ethanol is then recovered from the filtrate until no residual alcohol taste is detected, and the solution is concentrated to a relative density of 1.17 at 50°C before drying. Finally, the extracts are combined, ground into a fine powder, and mixed with dextrin to produce SLFM granules. According to the established quality standards, each gram of SLFM granule contains the equivalent of 2.25 grams of medicinal herbs.

### 2.7 HPLC-MS

The chemical constituents of SLFM were analyzed using an ACQUITY UPLC I-Class HF system coupled with a Q Exactive high-resolution mass spectrometer. Detailed procedures for sample preparation, chromatographic separation, and mass spectrometric detection are described in Supplementary Method 1.

### 2.8 Rats

Male Sprague-Dawley rats (aged 6–8 weeks, weighing 200 ± 20 g) were obtained from Shanghai Slake Experimental Animal Co., Ltd. They were housed in a specific pathogen-free (SPF) laboratory at the Experimental Animal Center of Zhejiang Chinese Medical University. The rats were maintained at 25 ± 2°C and 55% ± 5% relative humidity, under a 12-hour light-dark cycle, with *ad libitum* access to food and water. Experiments commenced after a one-week acclimatization period.

### 2.9 Langendorff- perfused working heart

The SD rats were weighed, euthanized with 3% pentobarbital sodium (50 mg/kg), and heparinized (3,125 U/kg). They were then placed on the experimental table, fixed, and subjected to a T-shaped thoracotomy to expose the heart. The lungs were clamped with forceps to elevate the heart, which was quickly excised along the posterior aspect of the lungs and placed in a glass petri dish containing pre-chilled liquid. The aorta was promptly located, excess tissue was trimmed away, and the aorta was carefully positioned at the bottom of the cannula, secured with surgical sutures. A pre-prepared KH solution [NaCl 119, KCl 4, CaCl_2_ 1.8, MgCl_2_ 1, KH_2_PO_4_ 1.2, NaHCO_3_ 25, and Glucose 10 (mM)] was gently injected into the heart using a syringe to remove residual blood, followed by Langendorff perfusion. The perfusion rate was set at 10 mL/min, and the temperature was maintained at 37 ± 0.5℃. Two separate electrodes were placed near the left ventricle (positive pole) and right atrium (negative pole) to record the ex vivo electrocardiogram.

### 2.10 Electrical mapping of isolated heart specimens

To stimulate the left atrium (LA), bipolar leads are employed. Given the S1S2 stimulation frequency, the atrial effective refractory period (AERP) can be calculated. Intracardiac and epicardial excitation mapping was conducted using a custom multi-electrode array comprising 64 electrodes in Langendorff-perfused isolated hearts. The standard for determining Activation Time (AT) is defined as the moment corresponding to the maximum rate of depolarization of the field potential (FP), as illustrated in isochronal maps. Meanwhile, the standard for determining Conduction Velocity (CV) is the speed measured from the earliest excitation point to the latest excitation point of the cardiac electrical signal, analyzed with EMapScope 5.0 software (MappingLab Ltd., UK).

### 2.11 Establishment of rat AF model and treatment protocol

The establishment of the AF model was accomplished using Ach (66 μg/mL) and CaCl_2_ (10 mg/kg), administered via tail vein injection at a dosage of 0.1 mL/100 g for five consecutive weeks, with drug intervention commencing one week later (14). The formula for converting equivalent doses based on body surface area between humans and animals, as outlined in “Pharmacological Experiment Methodology”, was referenced (15, 16). Utilizing the body surface area (BSA) normalization method (coefficient 0.018), the dosage calculation for rats is as follows: Rat dosage = Human dose (g/day) × 0.018÷0.2 kg. The clinical dosage of SLFM for treating AF is one sachet (6 g, with each 1 g of SLFM containing 2.25 g of crude drugs) to be taken three times daily. Consequently, the adult oral dose is calculated as 2.25 × 6 × 3 = 40.5 g/day. Thus, the dosage for rats is 40.5 g/day × 0.018÷0.2 kg = 3.645 g/kg/day. The high-dose group is established at twice the equivalent dose, amounting to 7.29 g/kg. Additionally, amiodarone was administered intragastrically to rats at a dosage of 0.18 g/kg (14). The rats were randomly divided into five groups and orally administered treatments for four weeks: a blank control group (distilled water), an AF group (distilled water), a low SLFM group (SLFM-L, 3.645 g/kg/day), a high SLFM group (SLFM-H, 7.29 g/kg/day), and an amiodarone group (0.18 g/kg/day).

### 2.12 Echocardiography

Echocardiographic monitoring was conducted using the Vevo 1100 small animal ultrasound system. Anesthesia was induced in rats with 3% isoflurane and maintained at a lower concentration of 1.5% ± 2%. Echocardiographic data were acquired from parasternal long-axis images. Key parameters measured included left ventricular ejection fraction (LVEF), left ventricular fractional shortening (LVFS), left ventricular internal dimension at systole (LVIDs), left ventricular internal dimension at diastole (LVIDd), interventricular septal thickness at systole (IVSs), interventricular septal thickness at diastole (IVSd), left ventricular posterior wall at systole (LVPWs), and left ventricular posterior wall at diastole (LVPWd). These measurements were obtained using two-dimensional echocardiography over five consecutive cardiac cycles. All measurements were taken with calipers only when ventricular systole and aortic valve leaflet tissue were clearly visible. By adjusting the probe angle, early diastolic blood flow peak velocity and atrial systolic blood flow peak velocity were measured from the apical four-chamber view to derive the E/A ratio. To evaluate each measurement point, the average value was calculated.

### 2.13 Intracardiac electrophysiological examination and induction of AF

Following the evaluation of echocardiography, 2% pentobarbital sodium (0.3 mL/100 g) was administered for intraperitoneal anesthesia in rats. Electrocardiogram (ECG) signals were recorded using Power Lab and LabChart 8. Electrodes were implanted in the subcutaneous tissue of the limbs, and surface ECG parameters were collected using limb lead II from each group, with a recording duration of 1 min for the ECG data. Subsequently, a 1/2-inch incision was made along the midline on the right side, with the tail end positioned at the level of the clavicle. After isolating the right jugular vein, it was ligated near the proximal end using a 6-0 suture. A small incision was made along the vein, and a 1.6 F octapolar electrode catheter (EPR-802; Millar) was inserted through the right jugular vein to record intracardiac electrograms. The inducibility of AF was tested three times following the protocol described by Verheule et al (17). An automatic stimulator, integrated with data acquisition software, was employed to apply 2-second bursts for testing the inducibility of ventricular arrhythmias. The cycle length (CL) of the initial 2-second burst was set at 40 ms, decreasing by increments of 2 ms to 20 ms in each subsequent burst. AF was defined as a rapid, irregular atrial rhythm lasting a minimum of 2 s. Electrode voltages were amplified using an eight-channel bioamplifier (FE238; ADInstruments) and recorded with LabChart 8 software (ADInstruments).

### 2.14 Plasma isolation and ELISA

Collect 6.0 mL of rat blood using an EDTA tube and store it at 4℃ for 24 h. Subsequently, centrifuge the sample at 3,000 rpm for 20 min to separate the plasma, which should then be stored at −80℃ for future analysis. Rat serum samples are analyzed for tumor necrosis factor-α (TNF-α, JL13202-96T, JONLNBIO), interleukin-1β (IL-1β, JL20884-96T, JONLNBIO), ras-related C3 botulinum toxin substrate 1(Rac1, JL15640-36T, JONLNBIO), and brain natriuretic peptide (BNP, JL11495-96T, JONLNBIO) levels using ELISA.

### 2.15 Histology and immunochemistry analysis

To evaluate the effects of SLFM on atrial morphology and structural remodeling in rats with AF, histopathological analyses of the left atrium were performed. After echocardiography and electrophysiological assessments, hearts were isolated and fixed in 4% paraformaldehyde for 24 h. Tissues were then dehydrated, embedded in paraffin, and sectioned at 5 μm thickness. Masson's trichrome staining was used to visualize collagen, and Terminal deoxynucleotidyl transferase-mediated dUTP nick end labeling (TUNEL) staining was performed to detect apoptosis. For immunohistochemistry, left atrial sections were incubated with anti-CX43 antibody (catalog number ab11370; Abcam). Imaging was performed using a Nikon Eclipse Ti-SR microscope, and ImageJ software was used to quantify integrated optical density (IOD) and the area of immunoreactive regions.

### 2.16 Statistical analysis

All datasets were tested for normality using the Shapiro–Wilk test and for homogeneity of variance using the Brown–Forsythe test. The incidences of AF (%) was analyzed using the Fisher' exact probability test. For comparisons between two groups: If the data followed a normal distribution and exhibited homogeneity of variance, a t-test was used. If the data followed a normal distribution but lacked homogeneity of variance, Welch's *t*-test was applied. If the data did not follow a normal distribution, the Wilcoxon rank sum test was used. For comparisons among multiple groups: If the data followed a normal distribution and exhibited homogeneity of variance, one-way ANOVA followed by Tukey's *post hoc* test was performed. If the data followed a normal distribution but lacked homogeneity of variance, Welch's one-way ANOVA was used. If the data did not follow a normal distribution, the Kruskal–Wallis test was applied. Statistical analyses were conducted using GraphPad Prism 9.0 software (GraphPad Software, San Diego, CA). Continuous variables are expressed as mean ± standard error (SE). The specific sample size (*n*) for each experiment is indicated in the corresponding figure legends.

## 3 Results

### 3.1 Potential drug target sites prediction

**Figure 1:**
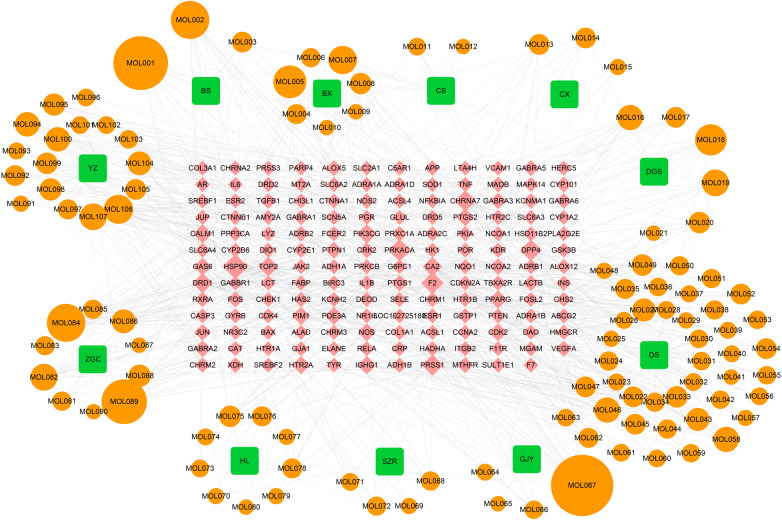
The TCM-compound-target network. BS *Baishao*, BX *Banxiaban*, CS *Chishao*, CX *Chuanxiong*, DGS *Dangshen*, DS *Danshen*, GJY *Guijianyu*, SZR *Suanzaoren*, HL *Huanglian*, ZGC *Zhigancao*, YZ *Yuanzhi*.

SLFM comprises 11 Chinese herbal medicines, namely *DanShen*, *BaiShao*, *Banxia*, *ChiShao*, *ChuanXiong*, *Dangshen*, *GuiJianYu*, *SuanZaoRen*, *Huanglian*, *ZhiGanCao*, and *YuanZhi*. Utilizing the TCMSP database, we identified key active ingredients, including *beta-sitosterol*, *Baicalin*, and *quercetin*, which exhibit high oral bioavailability and notable pharmacological properties (Supplementary Table S1). In total, 155 potential drug target sites were identified by predicting the potential targets of each active ingredient (Supplementary Table S2). The interrelationships among the herbs, active ingredients, and target sites are depicted (Figure 1), which encompasses 11 herbs, 107 effective active compounds, 155 potential target sites, and 855 associated clues.

### 3.2 Identification of key genes in AF

**Figure 2:**
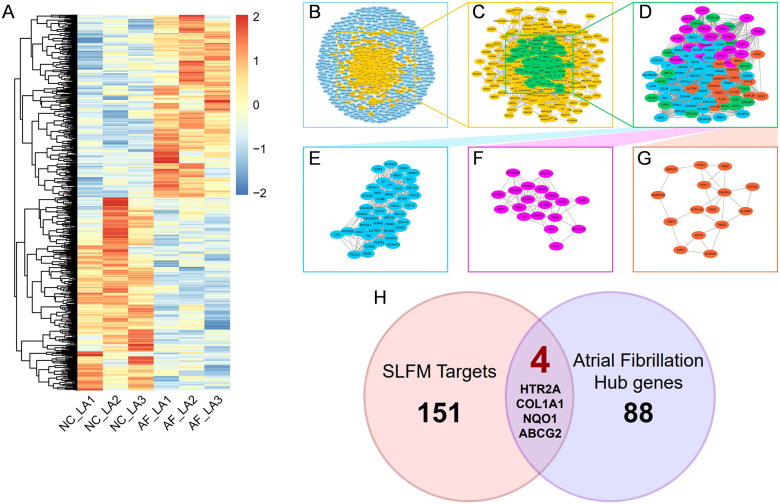
The process of discovering key intersected genes. **(A)** The heatmap of DEGs between normal individuals and patients with AF; **(B–G)** the PPI network analysis of the targets; **(H)** the Venn plot of SLFM and AF.

By comparing the left atrial appendage of patients with AF to that of a normal population, a total of 844 differentially expressed genes were identified. Among these, 410 genes were found to be upregulated in AF patients, while 434 genes were downregulated (Figure 2A). A protein-protein interaction (PPI) network of disease-relevant targets was constructed, comprising 4,088 reliable interaction relationships (Figure 2B). Given that drug action typically involves the simultaneous regulation of both upstream and downstream molecular mechanisms, 454 molecules isolated in the primary PPI network that did not interact with any other targets were excluded. This led to the construction of a secondary PPI network (Figure 2C). Additionally, the core network within the secondary PPI network was identified based on the number of connections, resulting in a tertiary PPI network containing 92 core targets, which are regarded as highly promising therapeutic candidates (Figure 2D). The MCODE plugin identified three major functional modules within the core network, which contained 42, 19, and 15 targets, respectively (Figures 2E–G). The Venn diagram illustrated that there were four overlapping genes between the potential target genes of SLFM and AF-related genes, thereby providing insights into the therapeutic potential of SLFM for treating AF (Figure 2H).

### 3.3 The action mechanism of core molecules related to AF

**Figure 3:**
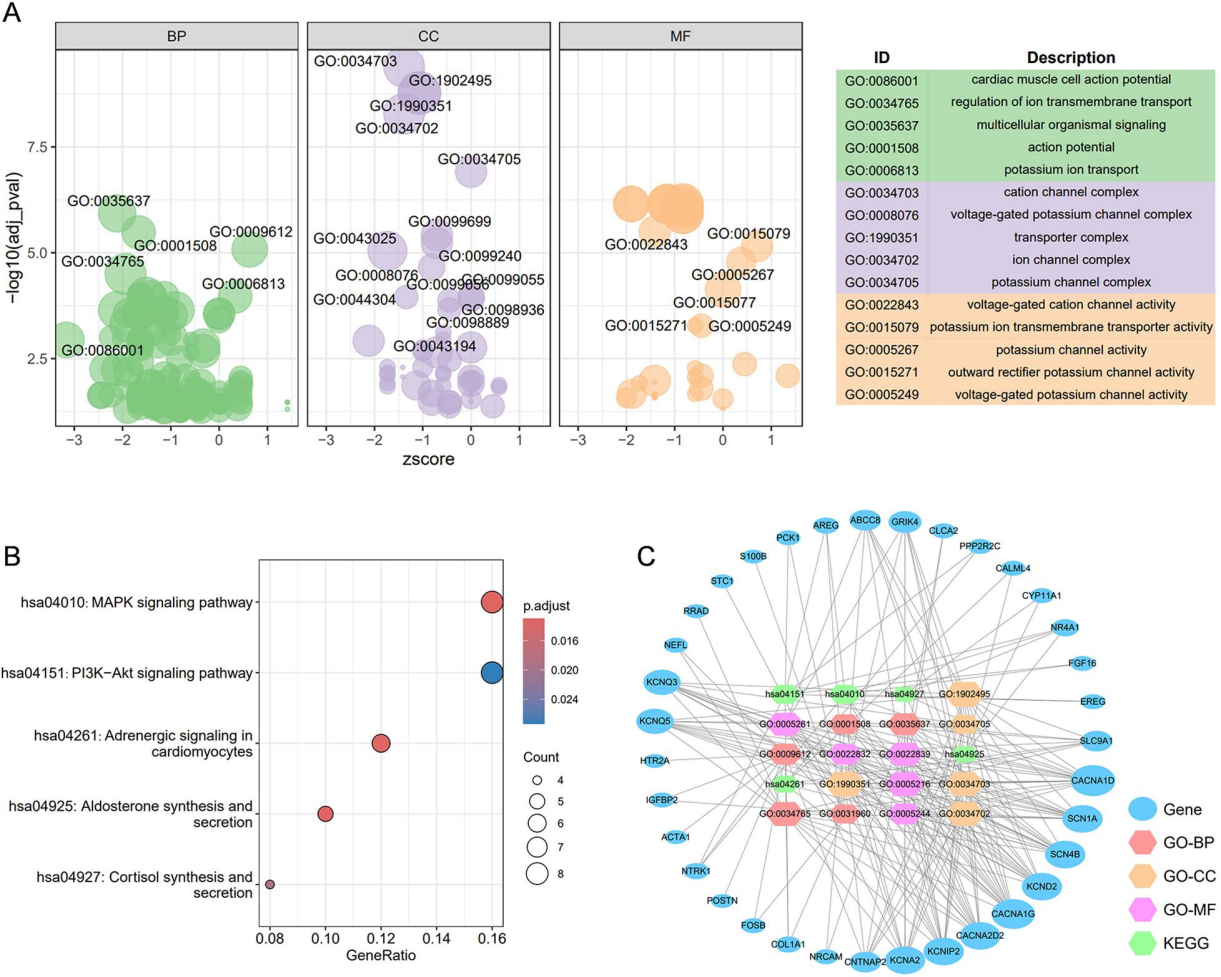
The construction of the target-function network. **(A)** The GO enrichment analysis for 92 core targets; **(B)** the top 5 KEGG enrichment analyses for 119 core targets; **(C)** the target-function network for the top 5 GO enrichment analysis and top 5 KEGG enrichment analyses. BP, biological process; CC, cellular component; MF, molecular function.

Enrichment analysis was performed to elucidate the action mechanisms of 93 core molecules associated with AF. GO enrichment analysis revealed a strong association between these core molecules and cardiac muscle cell action potential, a fundamental characteristic of AF, suggesting that the selected 93 molecules are highly reliable (Figure 3A). Additionally, the GO enrichment analysis indicated a significant relationship with cation signaling pathways, particularly potassium ion signaling pathways. KEGG enrichment analysis further demonstrated that these core targets are closely linked to the MAPK and PI3K-Akt signaling pathways (Figure 3B). Subsequently, we constructed a target-function network that visually represents the potential target molecules and their corresponding signaling pathway mechanisms (Figure 3C).

### 3.4 Interactions between key compounds and major target proteins

**Figure 4:**
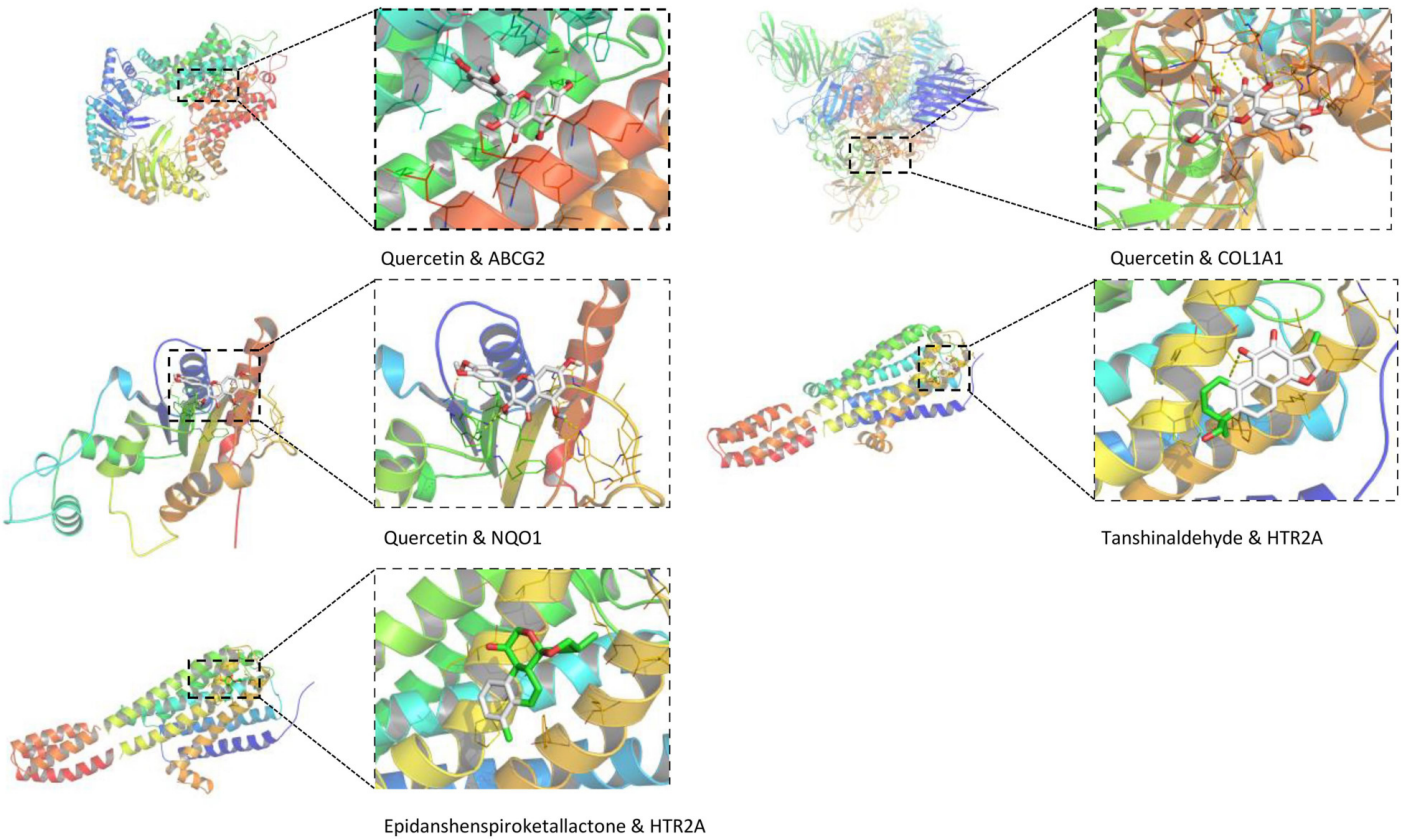
The conformations of main active compounds and major targets.

**Figure 5:**
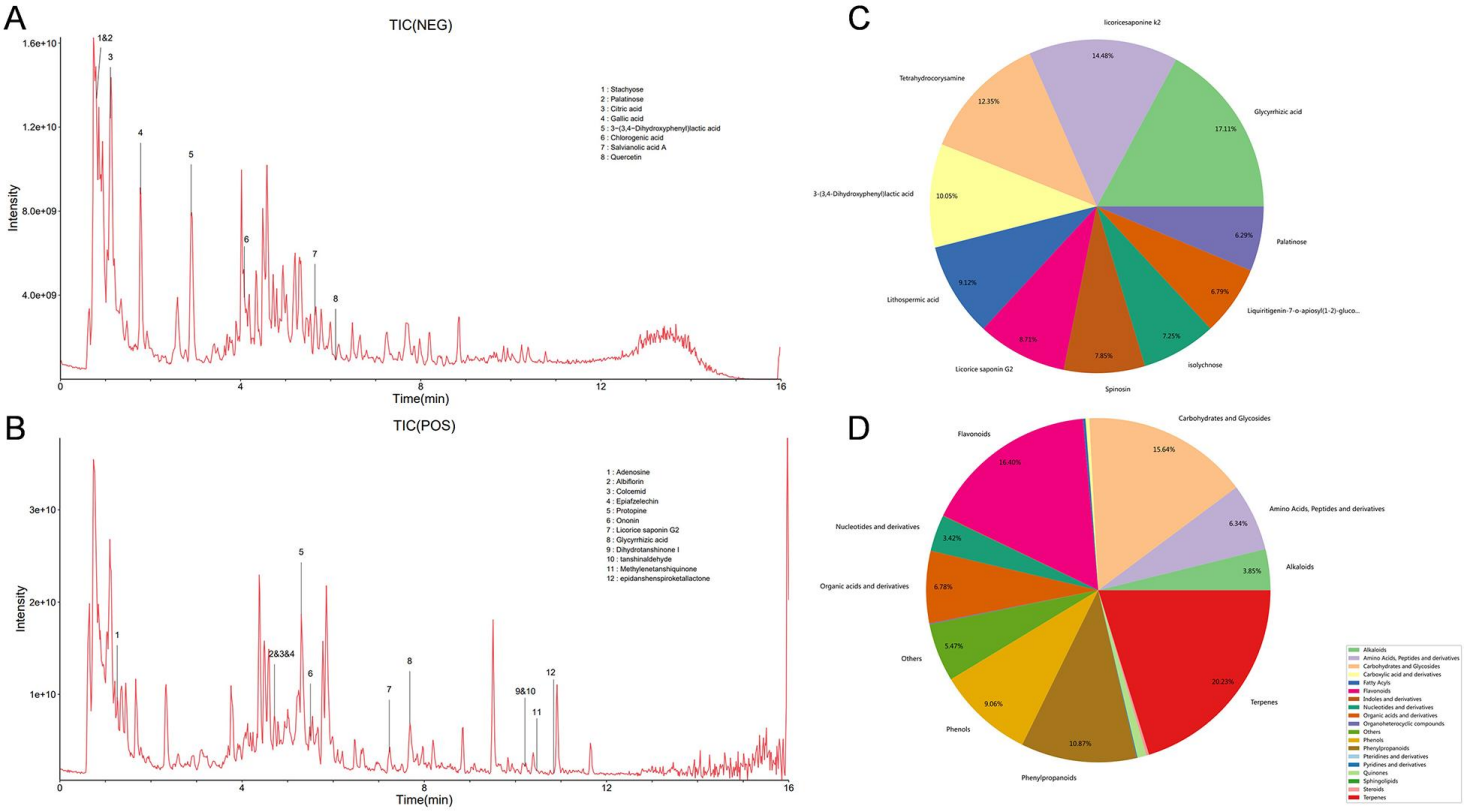
The HPLC-MS analysis for main components of SLFM. **(A)** Total ion chromatogram of SLFM extract in negative ion mode; **(B)** total ion chromatogram of SLFM extract in positive ion mode; **(C)** classification distribution chart of SLFM components; **(D)** composition classification quantity distribution chart of SLFM.

This study employs AutoDock Vina for molecular docking to investigate the interactions between key active compounds and major target proteins. The top three compounds, namely *quercetin*, *Tanshinaldehyde*, and *epidanshenspiroketallactone*, were docked with four pivotal target proteins: HTR2A, COL1A1, NQO1, and ABCG2 (Figure 4). Additionally, the binding energies for these five docking attempts are recorded and presented in Table 1, reflecting the affinity strength between the key compounds and the target proteins. To identify the primary components in SLFM and ensure quality control, we performed an analysis using HPLC-MS (Figure 5).

**Table 1:** The structure of the core compounds and binding energy between compounds and targets.

Docking item	Chemical structures of the active components in SLFM	Binding affinity/(kcal/mol)
Binding of *Quercetin* to the ABCG2	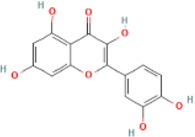	−7.9
Binding of *Quercetin* to the COL1A1		−5.2
Binding of *Quercetin* to the NQO1		−5.3
Binding of *Tanshinaldehyde* to the HTR2A	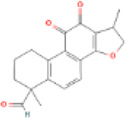	−5.7
Binding of *Epidanshenspiroketallactone* to the HTR2A	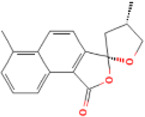	-5.5

### 3.5 ECG recording of isolated heart

The ex vivo electrocardiogram demonstrated that SLFM exerted minimal effects on heart rate, PR interval, *P* wave width, and QT interval in adult rats across a concentration range of 5–5,000 μg/mL (Supplementary Figure S1). Notably, we observed a trend toward QT interval shortening at concentrations between 5 μg/mL and 500 μg/mL, while a prolongation of the QT interval was noted at 5,000 μg/mL. These findings suggest that there is no risk of atrial or atrioventricular conduction block within the effective concentration range.

### 3.6 Electrical mapping of isolated heart specimens

The rat heart was isolated to perform epicardial mapping of the left atrium *in vitro*. Sixty-four-channel pen electrodes were employed to record atrial electrical signals under the rat's spontaneous rhythm, allowing for the analysis of atrial activation time (AT), conduction velocity (CV), and conduction dispersion. A reduction in conduction velocity can significantly contribute to reentry, a critical condition associated with AF. Our observations indicated that SLFM has a minimal impact on atrial activation time, conduction velocity, and conduction dispersion within the effective concentration range of SLFM (5–5,000 μg/mL) (Supplementary Figure S2A–D). Additionally, with S1S2 stimulation frequency, a concentration-dependent trend was noted, demonstrating a prolongation of the atrial effective refractory period (AERP) within the same effective concentration range of SLFM (5–5,000 μg/mL) (Supplementary Figure S2E-J), suggesting a potential anti-arrhythmic effect of SLFM.

### 3.7 The effect of SLFM on rat AF model assessed by echocardiography

**Figure 6:**
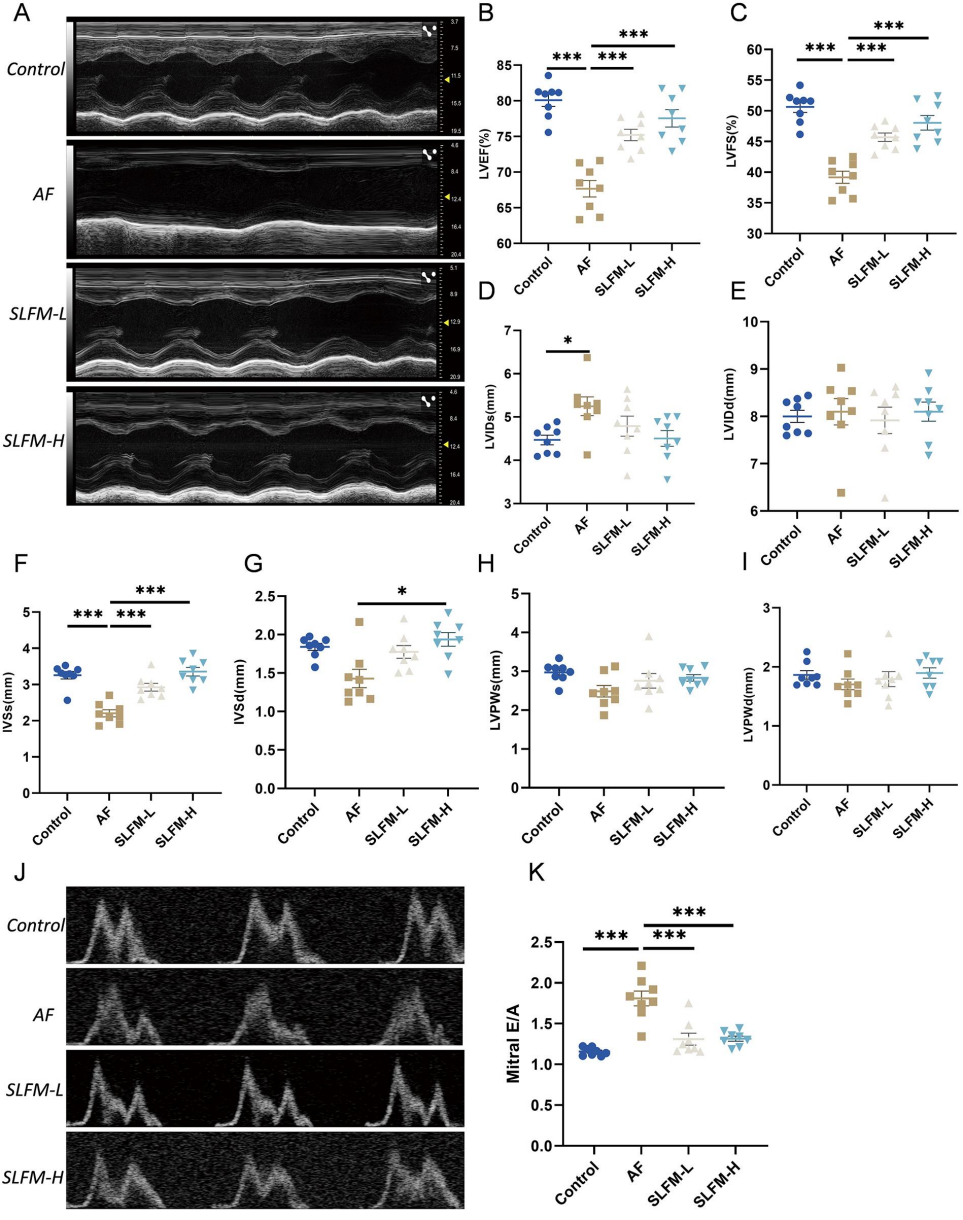
The effect of SLFM on Ach-CaCl_2_-induced AF in rats. **(A)** Representative M-Mode echocardiographic image; **(B–I)**: analysis results of LVEF, LVFS, LVIDs, LVIDd, IVSs, IVSd, LVPWs, and LVPWd; **(J)** Doppler recordings showing mitral valve flow; **(K)** quantitative E/A ratio. Data are expressed as mean ± SE (*n* = 8). * *p* < 0.05, ** *p* < 0.01, *** *p* < 0.001.

The analysis of echocardiography and hemodynamic parameters revealed a decrease in LVEF, LVFS, IVSs, and LVIDs in the AF group compared to the blank control group. There was no statistically significant difference observed in LVIDd, LVsd, LVPWs and LVPWd. Following treatment with SLFM-L and SLFM-H, LVEF, LVFS, and IVSs showed significant increases, while IVSd also increased after SLFM-H treatment, indicating that SLFM may enhance impaired ventricular function (Figures 6A–I). Furthermore, Doppler echocardiography indicated a reduction in the A wave in rats from the AF group, which resulted in an increased E/A ratio across the mitral valve. However, following treatment with SLFM-L and SLFM-H, the A wave exhibited recovery and the E/A ratio decreased, suggesting that SLFM could mitigate left ventricular diastolic dysfunction (Figures 6J,K).

### 3.8 SLFM reduced the inducibility and duration of AF in model rats

**Figure 7:**
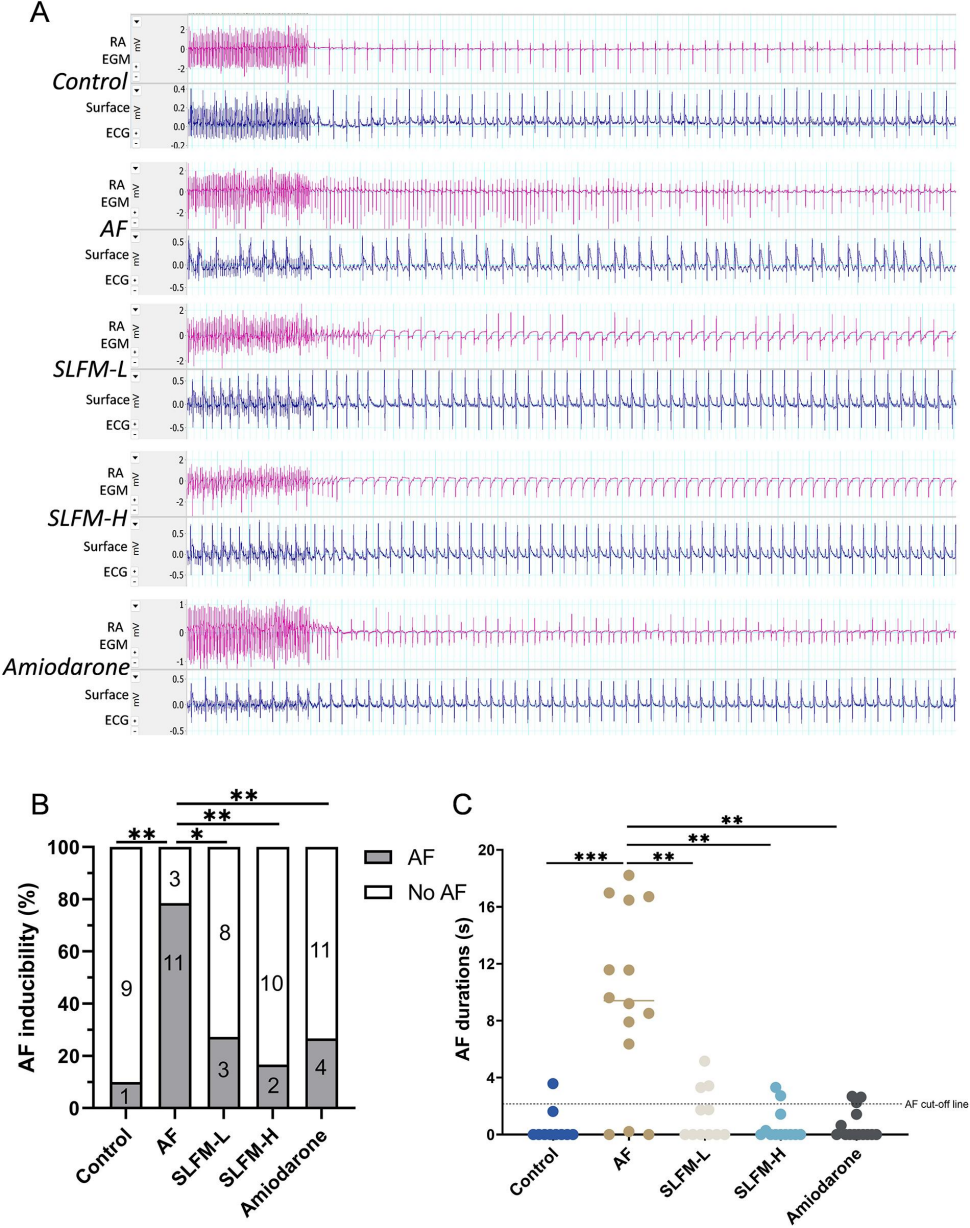
SLFM reduced the incidence and duration of AF induced in the rat model. **(A)** Typical electrocardiograms (lead II) and intracardiac electrograms were detected by programmed electrical stimulation to induce AF; **(B)** Incidence of pacing-induced AF in rats; **(C)** Durations of pacing-induced AF in rats. Data are expressed as mean ± SE (*n* = 10–15). * *p* < 0.05, ** *p* < 0.01, *** *p* < 0.001.

We further examined the basic electrocardiographic parameters and cardiac electrophysiological indices. The SLFM significantly reduced the occurrence of AF in rats (Figure 7B). Correspondingly, the duration of AF in the SLFM and amiodarone groups was significantly shorter than that observed in the AF group (Figure 7C). Based on these findings, SLFM demonstrates comparable efficacy to amiodarone in improving the electrophysiological characteristics in AF rats. Consequently, in the subsequent mechanistic experiments, only the SLFM-L and SLFM-H groups were compared and analyzed.

### 3.9 SLFM alleviated heart dysfunction, reduced inflammation, and inhibited fibrosis

**Figure 8:**
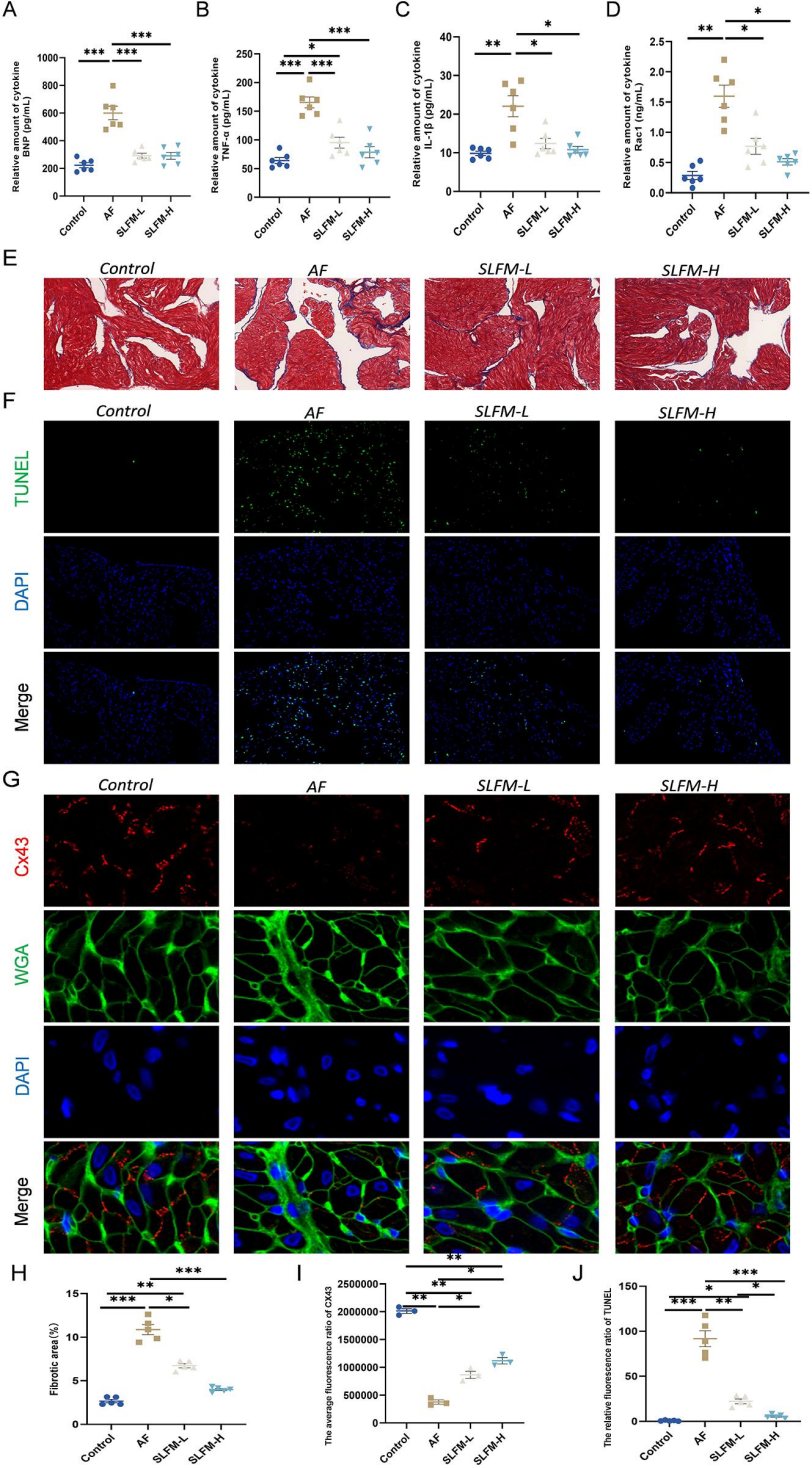
SLFM can alleviate heart dysfunction, reduce inflammation, and regulate structural remodeling. **(A–D)** The levels of serum TNF-α, IL-1β, Rac1, and BNP in rats were measured using the ELISA method; **(E)** Representative images of Masson staining (×40); **(F)** Representative image of TUNEL for detecting apoptosis (×200); **(G)** Representative images of immunofluorescence staining showing Cx43 (×400); **(H)** Quantitative ratio of fibrotic area to reference area; **(I,J)** Levels of Immunofluorescence in the left atrium of AF rats, including Cx43 and TUNEL. Data are expressed as mean ± SE (*n* = 3–6). * *p* < 0.05, ** *p* < 0.01, *** *p* < 0.001.

Inflammation plays a crucial role in the onset and progression of AF (18, 19). Consequently, we assessed the inflammatory response in the serum of various groups. The ELISA indicated that SLFM treatment reduced the levels of inflammatory factors TNF-α, IL-1β, and Rac1 (Figures 8B–D) compared to the AF group. Furthermore, echocardiography (Figures 6B,C) and ELISA of BNP (Figure 8A) demonstrated that SLFM could mitigate ventricular dysfunction. Additionally, atrial structural remodeling, characterized by fibrosis and atrial myocyte apoptosis, is considered critical for the onset and progression of AF (20–22). Masson's trichrome staining revealed that, relative to the blank control group, rats in the AF group exhibited significant atrial fibrosis, which was markedly reduced following SLFM treatment (Figures 8E,H). TUNEL staining of atrial tissues indicated a significant increase in TUNEL-positive cells in the AF group compared to the control group, with noticeable improvement after SLFM treatment (Figures 8F,J). Connexins, a type of ion channel protein, form gap junctions that facilitate communication between cells at the intercalated discs. These proteins possess high single-channel conductance, enabling cardiac tissue to function as an electrically continuous syncytium. The downregulation of Cx43, the predominant connexin in the atria, is closely linked to the development of AF (23, 24). Immunofluorescence staining revealed a decrease in the expression levels of gap junction protein Cx43 in the left atrial tissue of rats in the AF group, accompanied by redistribution among cells (Figures 8G,I), suggesting that SLFM could counteract the downregulation of Cx43 expression, with SLFM-H demonstrating a more pronounced effect.

## 4 Discussion

AF, as an age-related disease, affects nearly 20 million individuals in China (3). It significantly increases the risk of outcomes such as stroke and HF, resulting in high rates of disability and mortality with a poor prognosis (25). Modern medical treatment for AF typically employs methods such as radiofrequency ablation, AADs and surgical procedures. However, there are still some limitations regarding the recurrence of AF and the side effects and complications associated with medications, which can adversely affect clinical efficacy and prognosis. In recent years, TCM has demonstrated certain advantages in alleviating symptoms in patients with AF, enhancing patient survival and quality of life, as well as reducing postoperative adverse reactions.

In TCM theory, AF falls under the categories of palpitations and fright palpitations. Its fundamental pathogenesis encompasses both deficiency and excess aspects, which often intermix and transform into one another. Preliminary clinical studies have shown that SLFM can reduce the frequency of ventricular premature contractions in patients recorded by 24-hour dynamic electrocardiography, as well as alleviate symptoms of palpitations (11). Preliminary fundamental research indicates that SLFM can shorten the duration of action potentials induced by aconitine (26). This study builds upon prior research on Fumai-series formulas, which have demonstrated both electrophysiological and structural benefits in AF rodents. We aim to investigate the therapeutic effects of SLFM—a distinct formulation with 11 herbal components—on AF using network pharmacology, Langendorff perfusion, cardiac electrical mapping, and an Ach-CaCl₂-induced AF model, while also exploring its unique underlying mechanisms.

In SLFM, *quercetin*, *Tanshinaldehyde*, and *epidanshenspiroketallactone* have been identified as the top three significant active compounds. Research indicates that *quercetin* can ameliorate the pathological processes associated with AF (27).The enrichment analysis of the GEO database reveals a close association with cardiac muscle cell action potential, which is a fundamental characteristic of AF. Further analysis also indicates a close relationship with cation signaling pathways, particularly the potassium ion signaling pathway. The KEGG enrichment analysis indicates that these core targets are closely associated with the MAPK and PI3K−Akt signaling pathways. In our study, SLFM extended the effective refractory period of the atria in a dose-dependent manner within the concentration range of 5 μg/mL, 50 μg/mL, 500 μg/mL, and 5,000 μg/mL. Under sinus conditions, there was no significant change in the conduction velocity of the atria. A shortened effective refractory period and delayed conduction velocity can easily lead to the formation of reentry circuits, which are important conditions for the occurrence of AF. These results suggest that SLFM, with its unique multi-component profile, exerts anti-atrial arrhythmic effects, consistent with but distinct from other Fumai-series formulas.

In AF, the sustained activation of acetylcholine-dependent potassium currents (IK, ACh) is considered a key factor contributing to electrical cardioversion failure. Studies have shown that blocking IK, ACh can significantly reduce the atrial defibrillation threshold and improve the success rate of electrical cardioversion (28). Furthermore, research has revealed that during atrial remodeling caused by AF, the enhancement of acetylcholine-regulated potassium currents [I(KAChC)] correlates with shortened action potential duration and increased susceptibility to AF (29). Atrial myocytes are susceptible to Ca^2+^ concentration, and calcium overload can cause transient inactivation of calcium channels influenced by Ca^2+^ levels, reducing positive charge influx. This disrupts the effective depolarization of the action potential in atrial myocytes, leading to a shortened atrial effective refractory period, increasing atrial vulnerability to injury, and ultimately promoting the occurrence of AF (30).

To further investigate the mechanism of action of SLFM in the treatment of AF, we established an AF model through intravenous injection of Ach-CaCl_2_ for 5 weeks and administered SLFM via gavage for 4 weeks. The results from echocardiography indicated that the AF group experienced HF. AF can interfere with the heart's pumping function or blood accommodation, increase resting heart rate, lead to tachycardia, shorten left ventricular filling time, and subsequently decrease cardiac output, thus promoting the onset and progression of HF (31). However, after the intervention with SLFM, the HF in the rats showed significant improvement, and the secretion levels of BNP detected by ELISA corroborated this finding. The mechanism for the formation of the A wave at the mitral valve orifice is triggered by left atrial contraction; however, during AF, effective left atrial contraction pressure is difficult to generate. Consequently, it becomes challenging to create a pressure gradient between the left atrium and left ventricle to propagate the mitral valve and produce the A wave, resulting in a decreased E/A ratio. After pharmacological treatment, there is a significant improvement in the E/A ratio compared to the AF group.

To evaluate the effect of SLFM on the susceptibility of rats to AF, we conducted an electrophysiological examination and the induction of AF. Compared to the AF group, SLFM treatment reduced both the incidence and duration of AF. Additionally, it was found that SLFM can reduce the secretion of inflammatory factors. The pathophysiology of atrial remodeling in the context of inflammation and AF is closely intertwined. It is believed to be associated with the formation of substrates that promote AF, and as related immune responses occur, it participates in the onset and maintenance of AF (32). The explanation indicates that SLFM can play a therapeutic role in the treatment of AF through its anti-inflammatory effects. The structural remodeling of the atrium plays a significant role in the onset and maintenance of AF. This phenomenon has been observed in patients with AF, aging animal models, and those with congestive HF (33), which complicates the treatment of AF. Reports discovered significant differences in serum type I collagen (COL1) metabolism markers between patients with AF and elderly patients (34). Masson staining revealed that, compared to the AF group, SLFM inhibited collagen deposition in the atria and alleviated atrial fibrosis. During our molecular docking experiments, we also discovered that *quercetin* exhibits a strong affinity for COL1A1 (≤-5 kcal/mol), suggesting that the active components of SLFM can potentially treat or alleviate AF through this target. In the heart, connexins are responsible for maintaining rapid electrical conduction between cells, allowing for synchronized contractions. Cx43 is highly expressed in cardiomyocytes, and the downregulation of Cx43 expression is closely associated with the occurrence of atrial AF. The immunofluorescence results for Cx43 indicate that SLFM may prevent the occurrence of AF by regulating the expression of Cx43. A study found that treatment with the PI3K inhibitor (LY294002) can reduce the expression of Cx43 in diabetic models (35). The KEGG enrichment analysis suggests that the core targets in SLFM are closely related to the PI3K−Akt signaling pathway. We believe that SLFM may enhance the expression of Cx43 through the PI3K−Akt signaling pathway. Furthermore, during the process of atrial structural remodeling, the apoptosis of atrial myocytes plays a crucial role. This apoptosis leads to cardiac remodeling, primarily due to the rearrangement of adjacent myocardial cells (36). We utilized the TUNEL method to assess the apoptosis of atrial tissue in rats and found that SLFM effectively inhibits the apoptosis of atrial myocytes.

We acknowledge the limitations of the Ach-CaCl_2_ model in comprehensively replicating all clinical AF substrates. In future studies, we plan to incorporate additional models, such as rapid pacing or heart failure-induced AF, to further validate the broad applicability of the SLFM. Although our study has preliminarily revealed the potential of SLFM in suppressing AF by modulating atrial electrical and structural remodeling, its specific mechanisms at the ion channel level remain unclear. To further elucidate the direct electrophysiological effects of SLFM on atrial cardiomyocytes, we plan to employ whole-cell patch-clamp techniques in subsequent studies. This will allow us to systematically evaluate the impact of SLFM and its key active compounds (such as *quercetin*, *Tanshinaldehyde*, and *epidanshenspiroketallactone*) on action potential duration and major ion currents (e.g., IK-ACh, ICa-L, Ito, IKur). Furthermore, by combining gene knockout or siRNA interference approaches, we aim to verify whether SLFM exerts its antiarrhythmic effects through key targets (e.g., HTR2A, COL1A1, NQO1, ABCG2) and related signaling pathways (such as PI3K-Akt and MAPK). These investigations are expected to provide more robust experimental evidence at the cellular and molecular levels to support the clinical application of SLFM.

This study has initially explored the effects of SLFM in the treatment of AF and its possible mechanisms. However, the causal relationship between traditional Chinese medicine's anti-inflammatory effects and its role in suppressing AF has yet to be clarified, which requires further investigation. Additionally, the mechanisms underlying atrial fibrosis are complex, and the specific TCM responsible for the primary effect within the formula also necessitates further research for validation. TCM in Asia, particularly in China, has a rich history dating back nearly 3,000 years. The occurrence of AF is the result of a complex interplay of multiple factors, making it challenging to achieve effective treatment using single-target therapies. In contrast, the multi-target approach of TCM may offer a potential solution to this issue. Given that TCM are not widely used among patients with AF, our research findings provide new evidence for the therapeutic potential of SLFM as a TCM.

## 5 Conclusion

In summary, our research highlights the role of SLFM—a uniquely composed Fumai-series formula—in attenuating AF through multi-target mechanisms involving electrical and structural remodeling. Given the consistent therapeutic effects of SLFM observed in clinical practice, we utilized network pharmacology analysis to align the drug targets of SLFM with key molecules associated with AF. This analysis suggests that SLFM may participate in various pathways related to AF. In AF rat models, SLFM has been shown to inhibit atrial fibrosis and the inflammatory response, upregulate the expression of Cx43 to prevent both electrical and structural remodeling of the atria, and suppress the onset and progression of AF.

## Data Availability

The original contributions presented in the study are included in the article/Supplementary Material, further inquiries can be directed to the corresponding authors.

## Supplementary material

Supplementary Figure S1 The effects of different concentrations of SLFM on heart rate, PR interval, and QT interval in rats. **(A)** ECG representation graph; **(B)** Heart rate statistical graph; **(C)** PR interval statistical graph; **(D)** *P* wave width statistical graph; **(E)** QT interval statistical graph. Data are expressed as mean ± SE (*n* = 2).

Supplementary Figure S2 The effects of different concentrations of the drug SLFM on the electrophysiological conduction in the atria of rats, as well as the electrophysiological conduction in the left atrium of rats during S1S2 stimulation. **(A)** Schematic representation of cardiac mapping; **(B)** Representative diagram of left atrial conduction under sinus rhythm, conduction dispersion diagram; **(C)** Statistical diagram of left atrial conduction velocity and conduction dispersion under sinus rhythm; **(D)** Statistical diagram of right atrial conduction velocity and conduction dispersion under sinus rhythm. **(E–I)** represent the left atrial field potential and stimulation protocol representative graphs before and after drug administration during S1S2 stimulation; **(J)** The graph shows the statistical analysis of the AERP. Data are expressed as mean ± SE (*n* = 2).

Supplementary Table S1 Information for candidate bioactive compounds retrieved in SLFM.

Supplementary Table S2 155 potential drug target sites.
