# Supplementary figures and images for: The Shenlian Fumai Granule attenuates Ach-CaCl2-induced atrial fibrillation by regulating atrial electrical and structural remodeling
Source: Front Cardiovasc Med. 2025 Dec 15;12:1573728. doi: 10.3389/fcvm.2025.1573728 (PMC12745443; doi:10.3389/fcvm.2025.1573728)

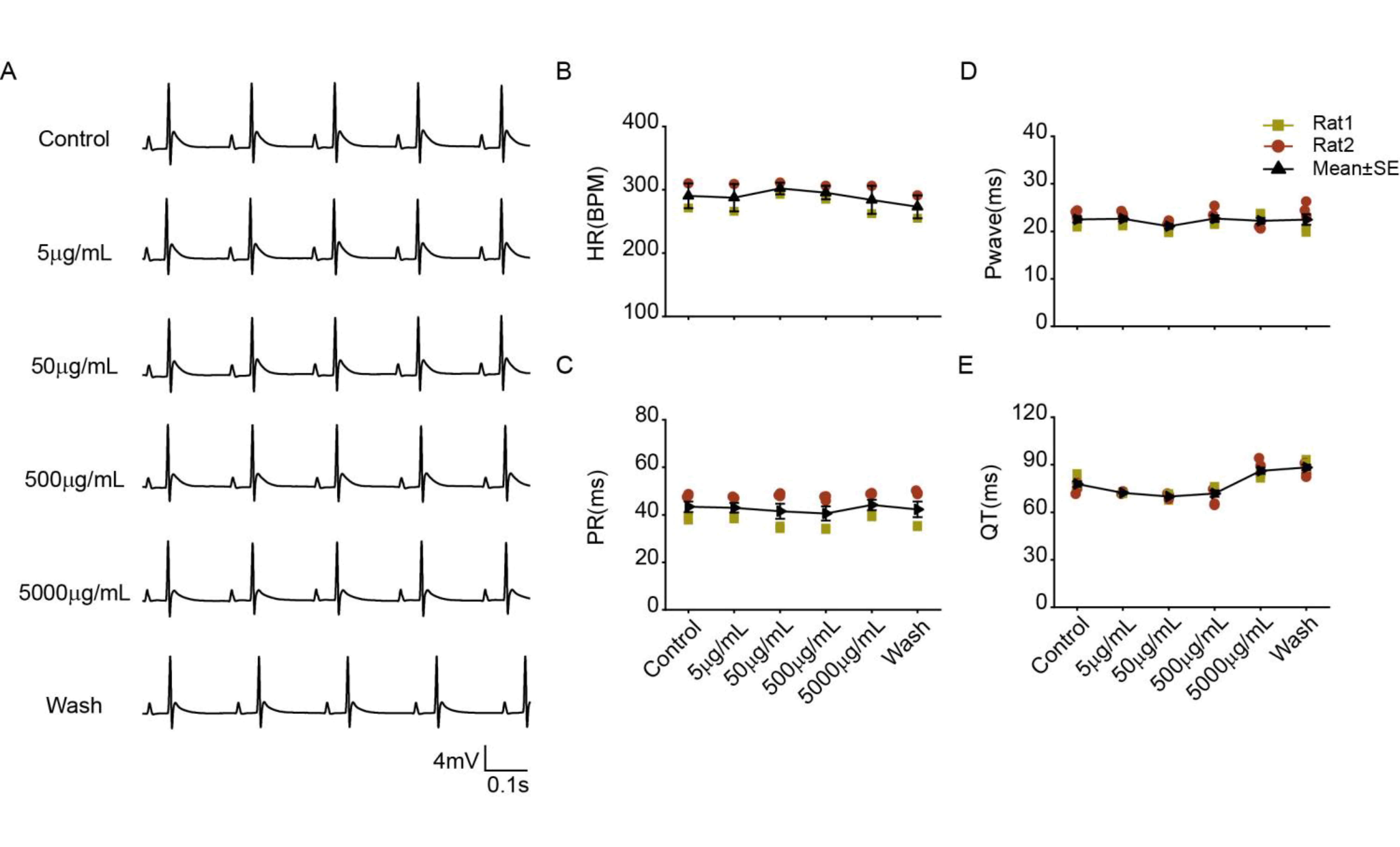

Supplement: Supplementary Figure S1 — The effects of different concentrations of SLFM on heart rate, PR interval, and QT interval in rats. (A) ECG representation graph; (B) Heart rate statistical graph; (C) PR interval statistical graph; (D) P wave width statistical graph; (E) QT interval statistical graph. Data are expressed as mean ± SE (n = 2). [file Image1.jpeg]

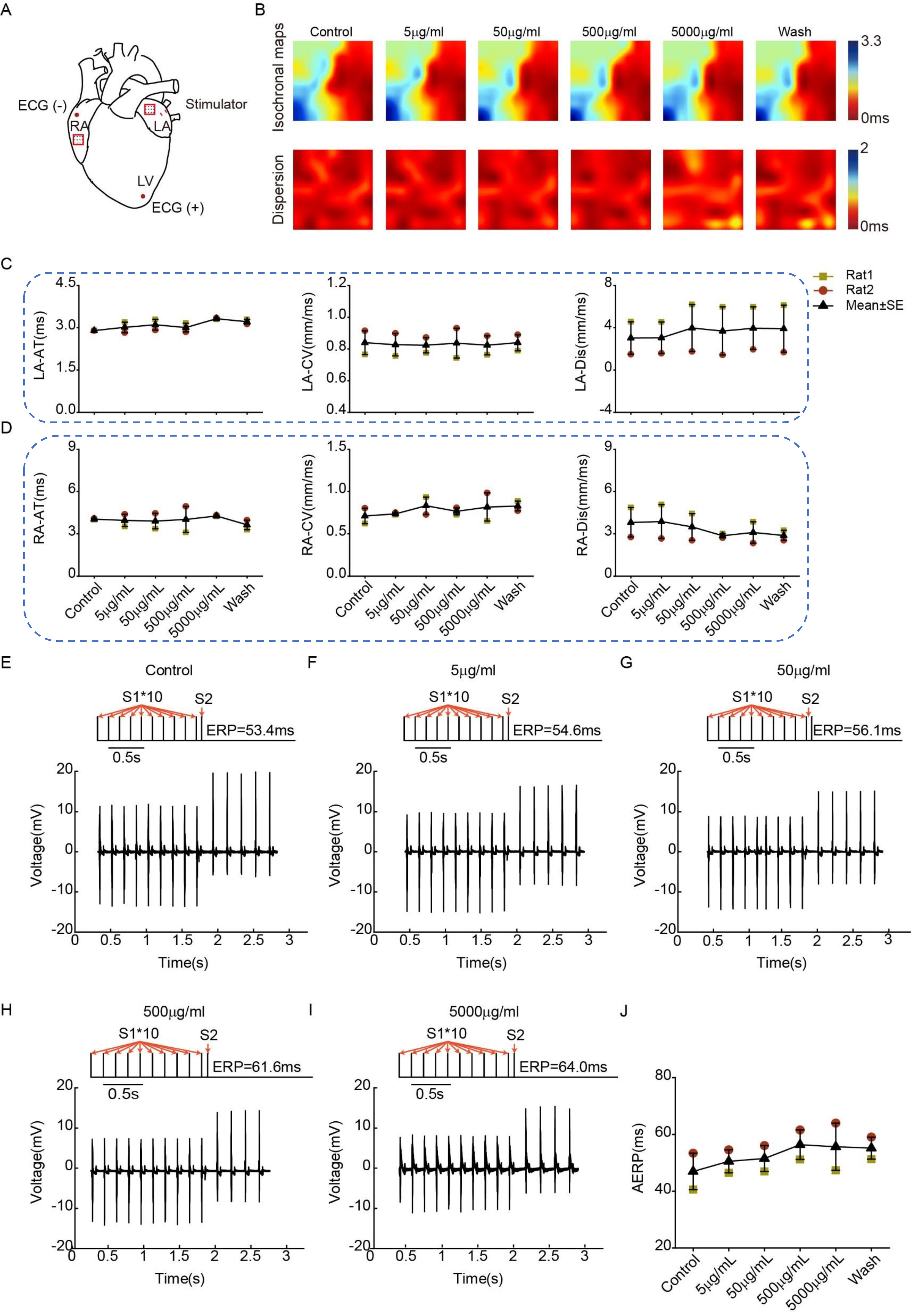

Supplement: Supplementary Figure S2 — The effects of different concentrations of the drug SLFM on the electrophysiological conduction in the atria of rats, as well as the electrophysiological conduction in the left atrium of rats during S1S2 stimulation. (A) Schematic representation of cardiac mapping; (B) Representative diagram of left atrial conduction under sinus rhythm, conduction dispersion diagram; (C) Statistical diagram of left atrial conduction velocity and conduction dispersion under sinus rhythm; (D) Statistical diagram of right atrial conduction velocity and conduction dispersion under sinus rhythm. (E–I) represent the left atrial field potential and stimulation protocol representative graphs before and after drug administration during S1S2 stimulation; (J) The graph shows the statistical analysis of the AERP. Data are expressed as mean ± SE (n = 2). [file Image2.jpeg]
