# Supplementary material for: The Shenlian Fumai Granule attenuates Ach-CaCl2-induced atrial fibrillation by regulating atrial electrical and structural remodeling
Source: Front Cardiovasc Med. 2025 Dec 15;12:1573728. doi: 10.3389/fcvm.2025.1573728 (PMC12745443; doi:10.3389/fcvm.2025.1573728)
Supplement: Supplementary Table S1 — Information for candidate bioactive compounds retrieved in SLFM. [file Table1.pdf]

Information for candidate bioactive compounds retrieved in SLFM

NO	Molecule name	MW	OB(%)	DL
BaiShao				
MOL001	*beta-sitosterol*	414.79	36.91	0.75
MOL002	*kaempferol*	286.25	41.88	0.24
MOL003	*(+)-catechin*	290.29	54.82	0.24
BanXia				
MOL004	*Cavidine*	353.45	35.64	0.8
MOL005	*baicalein*	270.25	33.51	0.2
MOL006	*Baicalin*	446.39	40.12	0.75
MOL007	*Stigmasterol*	412.77	43.82	0.75
MOL008	*coniferin*	314.41	31.1	0.32
MOL009	*(3S,6S)-3-(benzyl)-6-(4-hydroxybenzyl)piperazine-2,5-quinone*	310.38	46.88	0.26
MOL010	*beta-D-Ribofuranoside, xanthine-9*	284.26	44.71	0.2
MOL001	*beta-sitosterol*	414.79	36.91	0.75
ChiShao				
MOL011	*ellagic acid*	302.2	43.06	0.43
MOL012	*(2R,3R)-4-methoxyl-distylin*	318.3	59.98	0.29
MOL001	*beta-sitosterol*	414.79	36.91	0.75

MOL003	*(+)-catechin*	290.29	54.82	0.24
MOL005	*baicalein*	270.25	33.51	0.2
MOL006	*Baicalin*	446.39	40.12	0.75
MOL007	*Stigmasterol*	412.77	43.82	0.75
ChuanXiong				
MOL013	*Myricanone*	356.45	40.59	0.51
MOL014	*Perlolyrine*	264.3	65.94	0.27
MOL015	*FA*	441.45	68.96	0.7
DangShen				
MOL016	*7-Methoxy-2-methyl isoflavone*	266.31	42.56	0.19
MOL017	*Frutinone A*	264.24	65.9	0.34
MOL018	*luteolin*	286.25	36.16	0.24
MOL019	*3-beta-Hydroxymethyllenetanshiquinone*	294.32	32.16	0.4
MOL020	*glycitein*	284.28	50.47	0.23
MOL021	*11-Hydroxyrankinidine*	356.46	40	0.66
MOL007	*Stigmasterol*	412.77	43.82	0.75
MOL014	*Perlolyrine*	264.3	65.94	0.27
DanShen				
MOL022	*1,2,5,6-tetrahydrotanshinone*	280.34	38.75	0.36
MOL023	*sugiol*	300.48	36.11	0.28
MOL024	*Dehydrotanshinone II A*	292.35	43.76	0.4

MOL025	*digallate*	322.24	61.85	0.26
MOL026	*5,6-dihydroxy-7-isopropyl-1,1-dimethyl-2,3-dihydrophenanthren-4-one*	298.41	33.77	0.29
MOL027	*2-isopropyl-8-methylphenanthrene-3,4-dione*	264.34	40.86	0.23
MOL028	*3α-hydroxytanshinoneIIa*	310.37	44.93	0.44
MOL029	*(E)-3-[2-(3,4-dihydroxyphenyl)-7-hydroxy-benzofuran-4-yl]acrylic acid*	312.29	48.24	0.31
MOL030	*4-methylenemiltirone*	266.36	34.35	0.23
MOL031	*2-(4-hydroxy-3-methoxyphenyl)-5-(3-hydroxypropyl)-7-methoxy-3-benzofurancarboxaldehyde*	356.4	62.78	0.4
MOL032	*formyltanshinone*	290.28	73.44	0.42
MOL033	*Methylenetanshinquinone*	278.32	37.07	0.36
MOL034	*Przewaquinone B*	292.3	62.24	0.41
MOL035	*przewaquinone c*	296.34	55.74	0.4
MOL036	*(6S,7R)-6,7-dihydroxy-1,6-dimethyl-8,9-dihydro-7H-naphtho[8,7-g]benzofuran-10,11-dione*	312.34	41.31	0.45

MOL037	*przewaquinone f*	312.34	40.31	0.46
MOL038	*tanshinaldehyde*	308.35	52.47	0.45
MOL039	*Danshenol B*	354.48	57.95	0.56
MOL040	*Danshenol A*	336.41	56.97	0.52
MOL041	*Salvilenone*	292.4	30.38	0.38
MOL042	*cryptotanshinone*	296.39	52.34	0.4
MOL043	*dan-shexinkum d*	336.41	38.88	0.55
MOL044	*danshenspiroketallactone*	282.36	50.43	0.31
MOL045	*deoxyneocryptotanshinone*	298.41	49.4	0.29
MOL046	*dihydrotanshinlactone*	266.31	38.68	0.32
MOL047	*epidanshenspiroketallactone*	284.38	68.27	0.31
MOL048	*C09092*	286.5	36.07	0.25
MOL049	*isocryptotanshi-none*	296.39	54.98	0.39
MOL050	*Isotanshinone II*	294.37	49.92	0.4
MOL051	*Miltirone*	282.41	38.76	0.25
MOL052	*neocryptotanshinone ii*	270.35	39.46	0.23
MOL053	*neocryptotanshinone*	314.41	52.49	0.32
MOL054	*1-methyl-8,9-dihydro-7H-naphtho[5,6-g]benzofuran-6,10,11-trione*	280.29	34.72	0.37
MOL055	*prolithospermic acid*	314.31	64.37	0.31

MOL056	*(2R)-3-(3,4-dihydroxyphenyl)-2-[(Z)-3-(3,4-dihydroxyphenyl)acryloyl]oxy-propionic acid*	360.34	109.38	0.35
MOL057	*salvianolic acid j*	538.49	43.38	0.72
MOL058	*salviolone*	268.38	31.72	0.24
MOL059	*(6S)-6-hydroxy-1-methyl-6-methylol-8,9-dihydro-7H-naphtho[8,7-g]benzofuran-10,11-quinone*	312.34	75.39	0.46
MOL060	*Tanshindiol B*	312.34	42.67	0.45
MOL061	*Przewaquinone E*	312.34	42.85	0.45
MOL062	*tanshinone iia*	294.37	49.89	0.4
MOL063	*(6S)-6-(hydroxymethyl)-1,6-dimethyl-8,9-dihydro-7H-naphtho[8,7-g]benzofuran-10,11-dione*	310.37	65.26	0.45
MOL006	*Baicalin*	446.39	40.12	0.75
MOL018	*luteolin*	286.25	36.16	0.24
MOL019	*3-beta-Hydroxymethyllenetanshiquinone*	294.32	32.16	0.4
GuiJianyu				

MOL064	*(2R)-5,7-dihydroxy-2-(4-hydroxyphenyl)chroman-4-one*	272.27	42.36	0.21
MOL065	*ZINC04073977*	412.77	38	0.76
MOL066	*5,7-dihydroxy-2-(3-hydroxy-4-methoxyphenyl)chroman-4-one*	302.3	47.74	0.27
MOL067	*quercetin*	302.25	46.43	0.28
MOL001	*beta-sitosterol*	414.79	36.91	0.75
MOL002	*kaempferol*	286.25	41.88	0.24
SuanZaoRen				
MOL068	*(S)-Coclaurine*	285.37	42.35	0.24
MOL069	*sanjoinenine*	489.67	67.28	0.79
MOL070	*swertisin*	446.44	31.83	0.75
MOL071	*zizyphusine*	342.45	41.53	0.55
HuangLian				
MOL072	*berberine*	336.39	36.86	0.78
MOL073	*berberrubine*	322.36	35.74	0.73
MOL074	*epiberberine*	336.39	43.09	0.78
MOL075	*(R)-Canadine*	339.42	55.37	0.77
MOL076	*Berlambine*	351.38	36.68	0.82
MOL077	*Magnograndiolide*	266.37	63.71	0.19
MOL078	*palmatine*	352.44	64.6	0.65
MOL079	*coptisine*	320.34	30.67	0.86

MOL080	*Worenine*	334.37	45.83	0.87
MOL067	*quercetin*	302.25	46.43	0.28
ZhiGanCao				
MOL081	*18beta-glycyrrhetinic acid*	470.76	22.05	0.74
MOL082	*astragalin*	448.41	14.02	0.73
MOL083	*glycyrrhetinic acid*	470.76	22.05	0.74
MOL084	*isoliquiritigenin*	256.27	85.32	0.14
MOL085	*isoquercitrin*	464.41	1.85	0.76
MOL086	*liquiritigenin*	256.27	85.32	0.14
MOL087	*narcissin*	624.6	5.09	0.65
MOL088	*ononin*	430.44	11.52	0.77
MOL089	*rutin*	610.57	3.2	0.68
MOL090	*schaftoside*	564.54	4.68	0.82
YuanZhi				
MOL091	*1-Peroxyferolide*	338.39	17.38	0.35
MOL092	*alpha-pinene*	136.26	46.24	0.05
MOL093	*α-spinasterol*	412.77	42.97	0.75
MOL094	*benzoic acid*	138.13	30.14	0.02
MOL095	*carvacrol*	150.24	43.28	0.02
MOL096	*citral*	152.26	22.51	0.02
MOL097	*Harman*	182.24	33.09	0.09
MOL098	*harmine*	212.27	56.8	0.13

MOL099	*hyperin*	464.41	6.93	0.76
MOL100	*limonene*	136.26	39.84	0.02
MOL101	*linalool*	154.28	39.8	0.02
MOL102	*Norharman*	168.21	18.88	0.08
MOL103	*Norhyoscyamine*	275.38	60.34	0.16
MOL104	*Onjixanthone I*	302.3	79.15	0.29
MOL105	*quercitrin*	448.41	4.03	0.73
MOL106	*sucrose*	342.34	7.17	0.22
MOL107	*thymol*	150.24	41.47	0.03
MOL085	*isoquercitrin*	464.41	1.85	0.76
MOL089	*rutin*	610.57	3.2	0.68
